# Children living in the slums of Bangladesh face risks from unsafe food and water and stunted growth is common

**DOI:** 10.1111/apa.14281

**Published:** 2018-04-17

**Authors:** Ishita Mostafa, Nurun Nahar Naila, Mustafa Mahfuz, Manoj Roy, Abu S.G. Faruque, Tahmeed Ahmed

**Affiliations:** ^1^ Nutrition and Clinical Services Division International Centre for Diarrhoeal Disease Research Bangladesh Dhaka Bangladesh; ^2^ Lancaster Environment Centre (LEC) Lancaster University Lancaster UK

**Keywords:** Contaminated water, Food security, Hygiene practices, Malnutrition, Stunted growth

## Abstract

**Aim:**

This study investigated the microbial quality of food and water consumed by children in four slums in Dhaka, the capital of Bangladesh, together with the associated risk factors.

**Methods:**

This cross‐sectional study took place from December 2015 to May 2016 and focused on 360 children under the age of five. We recorded household food security, namely adequate food for a healthy life, socio‐economic and nutritional status, hygiene and feeding practices. Food and water samples were analysed.

**Results:**

We found that 63% of the children were malnourished and 58% were stunted. Yeast and moulds were detected in 86% of the food samples and coliforms in 73%. All the water samples were contaminated with faecal coliforms, yeasts and moulds and *Staphylococcus*. Food insecurity affected 83% of households. Children were twice as likely to be malnourished if they were born with a perceived low birthweight or their mothers did not wash their hands with soap after cleaning the child's bottom following defecation. Exclusively breastfed children were less likely to develop malnutrition.

**Conclusion:**

Children from the Dhaka slums were frequently stunted and malnourished and contaminated food and water was common. Integrated efforts are essential to create public awareness about hygiene.

**Abbreviations**
CI: Confidence Interval
LAZ: Length for age *z*‐score
MUAC: Mid‐upper arm circumference
OR: Odds ratio
WAZ: Weight for age *z*‐score
WHO: World Health Organization
WLZ: Weight for length *z*‐score

**Key notes**

- This study investigated the microbial quality of food and water consumed by 360 children under the age of five in slums in the capital of Bangladesh, together with associated risk factors.
- We found that 63% of the children were malnourished and 58% were stunted.
- All of the water samples were contaminated, and yeast and moulds were detected in 86% of the food samples and coliforms in 73%.

## Introduction

The first two years of a child's life are considered the most critical period with regard to meeting their nutritional requirements. During this time, the child has increased nutritional needs to support rapid growth and development 1. The World Health Organization (WHO) recommends exclusive breastfeeding for the first six months of life and continued breastfeeding throughout the second year of life, along with complementary food. According to the WHO, complementary foods are foods other than breast milk and infant formula that are introduced to infants around six months of age to provide optimum nutrition 2. This is especially relevant as undernutrition accounts for 3.5 million deaths and 35% of the disease burden among children under five years of age 3. This level is exceptionally high in South Asia. In Bangladesh, 26%, 25% and 17% of children aged less than two years are stunted, underweight and wasted, respectively 4.

Preparing complementary food without maintaining proper hygiene practices exposes the child to various enteropathogens 5. As a result, the incidence of food and waterborne infectious diseases like diarrhoea is highest among this age group, leading to negative impacts on their nutritional status 6. A study conducted in Nigeria reported that most diarrhoeal episodes occurred in children aged less than two, which corresponded with the weaning period 7. A similar study conducted in Bangladesh found that 40% of complementary food consumed by children was contaminated with *Escherichia coli,* and this was mainly attributable to poor food preparation techniques 5.

A high prevalence of contaminated complementary food leads to frequent diarrhoeal episodes of different levels, which may result in changes in the function and structure of the human gut mucosa. This condition, previously described as tropical enteropathy, is also referred to as environmental enteropathy and currently called environmental enteric dysfunction. It is increasingly acknowledged as an underlying cause of inadequate growth in young children, particularly stunting 8.

Low‐income settlements, especially slums, demonstrate the unique representation of child malnutrition in developing countries 9. In Bangladesh, particularly in large cities, slums fall into different categories: private, government acknowledged or informally established on government land. They show significant differences in terms of socio‐economic status and hygiene and sanitation practices. There are also differences between food preparation and consumption practices. Although a lot of emphasis is placed on hygiene and sanitation globally, the importance of food safety is largely ignored. This issue has rarely been addressed for slums, where the problem is believed to be acute. Efforts to decrease microbial contamination in food and water and improve the nutrition of young children may not succeed without the associated controls on exposure to food and waterborne pathogens. Against this backdrop, this study was designed to observe the differences across selected slum types. The overall aim was to yield a better picture and provide direction for future policymaking.

## Methods

### Site selection

This study took place in four slums, selected on the basis of land tenure arrangements in Dhaka City through a workshop involving local stakeholders 10. The study labelled these settlements using the first letter of their name (B, G, K and S) with an intention to preserve anonymity in compliance with ethical guidelines set by the research sponsor, host and collaborating institutions. The study also did not want to draw any kind of public attention to any specific settlements by disclosing the true extent of malnutrition and prevailing food insecurity status among the children living there. Of these slums, B and K were located in Mirpur area in the north‐western part of Dhaka City. The other two, G and S, were located in the western part of the city. A short overview of the slums is provided in Appendix A. Briefly, Slum B was originally a government resettlement colony that transferred to private ownership; Slum K was a squatter settlement on government land; Slum G was run by the city corporation for its low‐paid employees and Slum S was privately owned.

**Figure 1:**
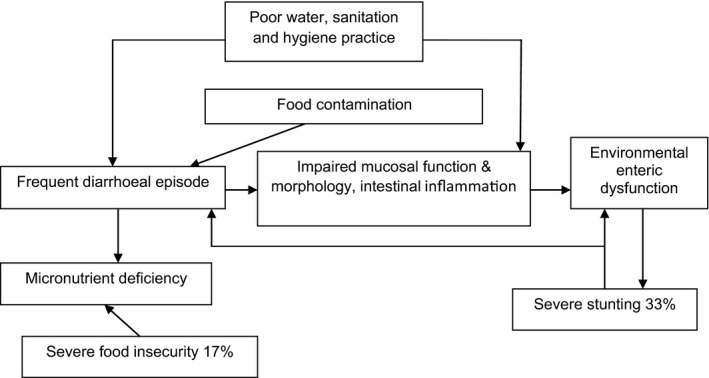
Inter‐relationship between study findings and environmental enteric dysfunction.

Children who were aged less than five years and living with their mothers or caregivers in one of the four slums of Dhaka city were eligible for this study. Children with chronic illness and congenital anomalies were excluded (Fig. 1).

### Sample size and sampling

**Figure 2:**
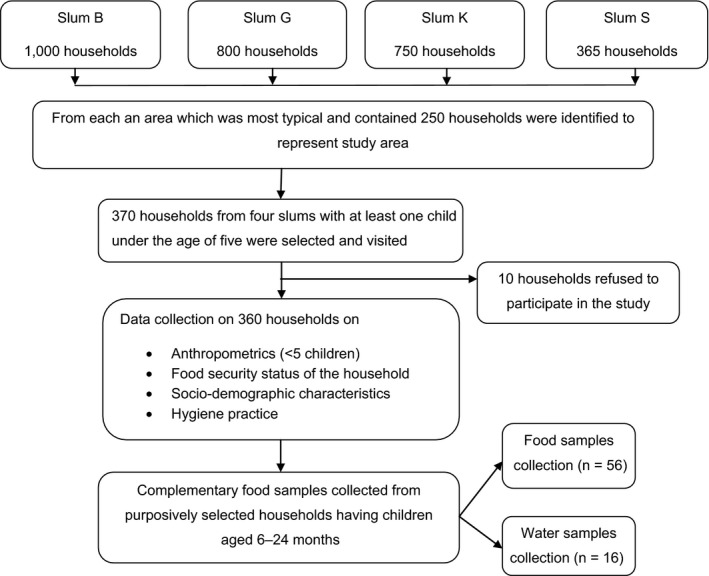
Flow chart showing sampling and subsampling procedure.

A sample size of 370 mothers or caregivers of children aged less than five years was estimated on the basis of 95% confidence interval (95% CI), at 0.05 level of desired precision, with a 41% prevalence of stunting based on similar contexts 4. A multistage sampling technique was followed for selecting the slums and study children. First, a list of slums was prepared by identifying slums that had at least 250 households and more than 1000 individuals. Stakeholders who took part in a workshop of stakeholders purposively identified four slums that differed in terms of number of residents, public or private ownership and the presence of common services and infrastructure. Of the 370 potential participants, 10 refused to take part in the study. The summary of sampling procedures is shown in Figure 2.

The Research Review Committee and Ethical Review Committee of the International Centre for Diarrhoeal Disease Research, Bangladesh, approved the study. Written, informed consent was obtained from all parents before the children were included in the study.

### Questionnaire and sample collection

A mixed, open and closed‐ended questionnaire was developed based on the objectives of the study, and the mothers or caregivers of the children were interviewed to obtain information on food insecurity, socio‐economic characteristics and hygiene practices. To assess the level of food insecurity, the questionnaire developed by Food and Nutrition Technical Assistance was used, which had already been validated and adapted for low resource settings. Further details are provided in Appendix A. The socio‐economic status of the households was assessed by the level of maternal education and monthly family income. The assessment of child's birthweight was based on the mothers’ perceptions about their size when they were born. A food sample questionnaire was administered to 72 mothers or primary caregivers who were responsible for the care of children under the age of two years, because complementary feeding is introduced to the infants and young children of this age group 5. The study purposively collected food samples from 56 children from the first 14 households from each of the four slums that were visited and could provide food samples. A total of 16 water samples, four from each of the four slums, were collected and sent to the Environmental Microbiology Laboratory of the International Centre for Diarrhoeal Disease Research, Bangladesh, following standard field specimen collection and transportation procedures.

### Anthropometry and microbial contamination of food and water

Body weight was measured to the nearest 1 g using the Dual Purpose Baby Scale (Seca, West Midlands, UK), and the mid upper arm circumference (MUAC) was measured using an MUAC tape. Standing height was measured to the nearest 0.1 cm using a Stadiometer, and supine length was measured to the nearest 0.1 cm using an Infantometer, also from Seca. Height for age (HAZ), weight for age (WAZ) and weight for height (WHZ) z‐scores were calculated using the WHO Anthro 2005 software to assess the nutritional status of the children. The WHO diagnostic criteria for severe acute malnutrition in children aged six to 59 months included any of the following: a WHZ below –3 SD of the WHO child growth standards, the presence of bilateral pedal oedema and an MUAC of <115 mm 11.

Aliquoting was carried out to identify Coliforms, *Escherichia coli*,* Bacillus cereus*, coagulase positive *Staphylococci* and yeasts and moulds. In order to isolate and identify the organisms, the same amount of samples were taken and mixed with selective enrichment media before being inoculated onto selective plating media 5.

For the quantitative analysis of the water, the enrichment cultures were inoculated onto selective plating media and the duration and temperature of the organisms were maintained according to the standard procedure. Typical colonies on culture plates were identified using standard procedures 12. See Appendix B for further details.

### Statistical analysis

Data collected from the field were coded and visually checked for errors before entering into a database. Computer coding was pretested, and the data entry and analyses were carried out using SPSS Statistics for Windows, version 20.0 (IBM Corporation, New York). All data were validated by a series of logical and range checks, and the statistical analyses included descriptive and analytic methods. A logistic regression, backward stepwise model was fitted to identify the factors that were considered significantly associated with malnutrition. A number of independent variables were used, including the age of the child, gender of the child, how many years the mother spent in education and whether they used soap to wash their hands before preparing food and after cleaning their child's bottom following defecation. We also included the family income, source of drinking water and household food security, which is having adequate food for a health life. The association between the nutritional status of the child and the mother's knowledge of hygiene practices was analysed in separate multivariate models. The variables were divided into groups on the basis of *a priori* logical categories, and proportions of different parameters were measured.

## Results

Data on 360 subjects were analysed for anthropometry, food security and the hygiene practices of the participants. Regular hand washing with soap after cleaning the child's bottom following defecation was higher than hand washing before preparing complementary foods (33% vs. 5%; p < 0.001). In the slums, the majority (71%) of families used water from public taps and 27% of households had water that was piped into their dwellings while a very few used filtered water (2%). Most of the families (76%) did not treat water before drinking and toilet sharing was a common practice (94%). Around 70% of the mothers used the same pot for storing food and feeding their children (data not presented). Most of the mothers (82%) prepared their children's food at home, while the others bought food from nearby small‐scale food establishments or street vendors. Most of the families stored their food at room temperature and only 1% refrigerated their food. The mean parental monthly income was 146 US dollars (standard deviation 73). More than 95% of the caregivers were married (Table 1), and more than six in 10 women (68%) were below 18 years of age at the time of marriage (data not presented).

**Table 1:** Characteristics of enrolled families and their under‐five children from four slums of Dhaka city, Bangladesh, December 2015 to May 2016

Variable (n = 360)	n = 360 (%)	95% CI	B n = 90 (%)	G n = 90 (%)	K n = 90 (%)	S n = 90 (%)
Socio‐economic status						
Income of household head in USD						
(mean ± SD)	145 ± 73		189 ± 78	151 ± 84	124 ± 48	115 ± 53
Education of mother/caregiver of the child						
No formal education	116 (32.0)	0.28–0.38	25 (27.8)	22 (24.5)	34 (37.4)	35 (38.8)
Primary incomplete	125 (35.0)	0.29–0.39	29 (32.2)	31 (34.4)	34 (37.8)	31 (34.4)
Completed primary	61 (17.0)	0.14–0.22	11 (12.2)	19 (21.1)	16 (17.8)	15 (16.7)
Secondary incomplete	44 (12.0)	0.09–0.17	18 (20.0)	14 (15.6)	5 (5.6)	7 (7.8)
Completed secondary	14 (4.0)	0.03–0.07	7 (7.8)	4 (4.4)	1 (1.1)	2 (2.2)
Hygiene status of mother or caregiver of the child						
Wash hands with soap after cleaning bottom of child following defecation						
Always	118 (33.0)	0.29–0.39	45 (50.0)	22 (24.4)	24 (26.7)	27 (30.0)
Sometimes	165 (46.0)	0.41–0.52	36 (40.0)	43 (47.8)	40 (44.4)	46 (51.1)
Rarely	50 (14.0)	0.12–0.18	2 (2.2)	18 (20.0)	19 (21.1)	11 (12.2)
Never	27 (8.0)	0.06–0.12	7 (7.8)	7 (7.8)	7 (7.8)	6 (6.7)
Wash hands with soap before feeding						
Always	20 (5.0)	0.04–0.09	3 (3.3)	10 (11.1)	7 (4.4)	3 (3.3)
Sometimes	86 (23.0)	0.19–0.29	13 (14.4)	24 (26.7)	32 (35.6)	17 (18.9)
Rarely	40 (11.0)	0.08–0.15	9 (10.0)	13 (14.4)	13 (14.4)	5 (5.6)
Never	214 (59.0)	0.55–0.64	65 (72.2)	43 (47.8)	41 (45.6)	65 (72.2)
Source of water						
Piped into dwelling	98 (27.0)	0.23–0.33	5 (5.6)	90 (100.0)	–	3 (3.3)
Public tab/stand pipe	255 (71.0)	0.66–0.76	85 (94.4)	–	90 (100.0)	80 (88.9)
Filter water	7 (2.0)	0.01–0.05	–	–	–	7 (7.8)
Drink boiled or treated water						
Yes	88 (24.0)	0.23–0.29	23 (25.6)	6 (6.7)	5 (5.6)	54 (60.0)
Toilet sharing						
Yes	340 (94.0)	0.92–0.97	70 (77.8)	90 (100.0)	90 (100.0)	90 (100.0)
Feeding practice						
Complementary food prepared at home	46 (82.0)	0.72–0.92	11 (12.2)	13 (14.4)	9 (10.0)	13 (14.4)
Complementary food bought from outside	10 (18.0)	0.08–0.28	3 (3.3)	1 (1.1)	5 (5.6)	1 (1.1)
Use of same pot for feeding and storing of Complementary food	54.0 (75.0)	0.65–0.85	14 (77.7)	16 (88.8)	11 (61.1)	16 (88.8)
Storing of food						
At room temperature	71.0 (99.0)	0.96–1.0	72 (100)	71 (99.0)	72 (100)	72 (100.0)
Refrigerator	1.0 (1.0)	0.01–0.08	–	1.0 (1.0)	–	–

1 USD = 78 BDT.

### Food and water sample contamination

All 56 food samples were universally contaminated with different microorganisms (Table 2). The most commonly detected microorganisms were yeast and mould (86%) and coliforms (73%), whereas *Vibrio cholera* was only detected in a few samples. Other detectable organisms were *Bacillus cereus* (48%), *Escherichia coli* (30%) and *Staphylococcus* (14%). On the other hand, *Salmonella* and *Shigella* were not found in any food samples. In the case of the water samples, total coliforms, faecal coliforms, total aerobic bacteria, *Staphylococcus* and yeast and mould were present in all 16 samples. More than half of the water samples were contaminated with *Escherichia coli* (63%) and faecal *Streptococci* (56%). There were no *Vibrio cholera*,* Shigella* or *Salmonella* in any of the water samples tested.

**Table 2:** Detection of foodborne pathogens in food and household water samples collected at point of use from four slums of Dhaka city, Bangladesh, December 2015 to May 2016

Organisms present in Food	Overall n = 56 n (%)	95% CI
Yeast and mould (>100 CFU/mg)	48.0 (85.7)	0.74–0.93
Coliforms (>100 CFU/mg)	41.0 (73.2)	0.59–0.84
*B. cereus* (>100 CFU/mg)	27 .0 (48.2)	0.35–0.62
*E. coli* (>100 CFU/mg)	17.0 (30.4)	0.19–0.44
*Staphylococcus* (>100 CFU/mg)	8.0 (14.3)	0.08–0.27
*V. cholera*	2.0 (3.5)	0.01–0.14

Organisms present in Water	Overall n = 16 n (%)	95% CI
Total coliforms	16.0 (100)	–
Faecal coliforms	16.0 (100)	–
Total aerobic bacterial count	16.0 (100)	–
Yeast	16.0 (100)	–
Mould	16.0 (100)	–
*Staphylococcus*	16.0 (100)	–
*E. coli*	10.0 (62.5)	0.35–0.86
*Faecal streptococci*	9.0 (56.3)	0.29–0.79
*Pseudomonas*	7.0 (43.8)	0.21–0.71

Total coliforms and faecal coliforms count (CFU/g).

The most commonly consumed foods by children were rice (59%) and vegetable curry (29%). They also received the following on an infrequent basis: tea, pitha cakes made with rice flour and sugar, parata flat breads made with whole‐wheat dough and oil, cake and biscuits (Table 3).

**Table 3:** Types of complementary foods collected at point of use from four slums of Dhaka city, Bangladesh, December 2015 to May 2016

Types of complementary food on site in urban slum children between 6–24 months of age (n = 56)	
Name of complementary foods	Number of collected spot samples of complementary foods n = 56 (%)
Rice	33 (58.9)
Vegetable curry	16 (28.5)
Lentil	12 (21.4)
Suji	10 (17.8)
Fish curry	9 (16.7)
Bread	6 (10.7)
Potato	5 (8.9)
Tea	5 (8.9)
Milk	5 (8.9)
Cake	4 (7.1)
Meat	3 (5.3)
Parata	2 (3.5)
Pitha	2 (3.5)
Biscuit	2 (3.5)
Chicken curry	1 (1.7)
Egg curry	1 (1.7)

The mean age of the study children varied from 26 months to 32 months across the four slums (Table 4). The highest rate of stunting (66%), wasting (12%) and underweight (44%) was found in Slum S (data not presented). The children in Slum S also demonstrated a higher proportion of undernutrition (11%), as assessed by an MUAC measurement of less than 12.5 cm. The proportion of children who were exclusively breastfed varied from 27% to 39% among the infants from the four slums, and the lowest rates were reported in Slum B and Slum S (27%).

**Table 4:** Demographic, nutritional status and breastfeeding practices, food security status of study children from four slums of Dhaka city, Bangladesh, December 2015 to May 2016

Characteristics	n = 360 (%)	B n = 90 (%)	G n = 90 (%)	K n = 90 (%)	S n = 90 (%)
Child's age in months					
mean (±SD)	29 ± 16.24	26 ± 18	30 ± 15	32 ± 14	27 ± 16
Male Children	174 (48.3)	34 (37.8)	40 (44.4)	54 (60)	46 (51.1)
Childs’ size at birth					
Smaller than average	82 (22.8)	20 (22.2)	15 (16.7)	25 (27.8)	22 (24.4)
Nutritional status					
Moderate underweight	85 (23.6)	22 (24.4)	21 (23.3)	19 (21.1)	23 (25.6)
Severe underweight	48 (13.3)	5 (5.6)	9 (10.0)	17 (18.9)	17 (18.9)
Moderate wasted	22 (6.1)	4 (4.4)	6 (6.7)	6 (6.7)	7 (7.8)
Severe wasted	5 (1.4)	0 (0.0)	1 (1.1)	1 (1.1)	4 (4.4)
Moderate stunted	89 (24.7)	19 (21.1)	28 (31.1)	20 (22.2)	22 (24.4)
Severe stunted	119 (33.1)	20 (22.2)	30 (33.3)	32 (35.6)	37 (41.1)
Malnourished	228 (63.3)	47 (52.2)	62 (68.9)	55 (61.1)	64 (71.1)
MUAC <12.5 cm	21 (5.8)	8 (8.9)	2 (2.2)	1 (1.1)	10 (11.1)
Exclusively breastfed	112 (31.1)	24 (26.7)	35 (38.9)	29 (32.2)	24 (26.7)
Food security					
Food secure	63 (17.5)	17 (18.9)	20 (22.2)	15 (16.7)	11 (12.2)
Mildly food insecure	65 (18.1)	19 (21.1)	24 (26.7)	19 (21.1)	3 (3.3)
Moderately food insecure	172 (47.8)	41 (45.6)	41 (45.6)	37 (41.1)	53 (58.9)
Severely food insecure	60 (16.7)	13 (14.4)	5 (5.6)	19 (21.1)	23 (25.6)

Moderate underweight −3 ≤ WAZ<‐2, severely underweight WAZ<‐3.

Moderate wasted −3 ≤ WHZ<‐2, severely wasted WHZ<‐3.

Moderate stunted −3 ≤ LAZ<‐2, severely stunted LAZ<‐3.

The food security status of the participating families is presented in Table 4. About 18% of the mothers reported their houses to be food secure. Severe food insecurity was highest in Slum S (26%) and lowest in Slum G (6%). A logistic regression model was constructed to determine the factors that were associated with the children's nutritional status. Hand washing with soap after cleaning the child's bottom following defecation, exclusive breast‐feeding practices and perceived normal birthweight were associated with better nutritional status. Children whose mothers did not practice hand washing with soap after cleaning the child's bottom following defecation were two times more likely to suffer from malnutrition with an odd ratio (OR) of 2.04 (95% CI 1.27–3.29). Similarly, children born with perceived low birthweight were two times more likely to be malnourished (OR 1.96; 95% CI 1.11–3.47). On the contrary, exclusively breastfed children were less likely to develop malnutrition (OR 0.44, 95% CI 0.27–0.70). Another logistic regression analysis that aimed to examine the factors associated with how many years of schooling the mothers received, demonstrated that poor household income (OR 2.88; 95% CI 1.70–4.89) was associated with low levels of education. In addition, poor hygiene practices, evidenced by not using soap after cleaning the child's bottom following defecation (OR 1.70; 95% CI 0.99–2.93) was marginally associated with low levels of education (p = 0.054) (Table 5).

**Table 5:** Logistic regression model showing relationship between independent variables and dependent variable of interest in under‐five children from four slums of Dhaka city, Bangladesh, December 2015 to May 2016

Dependent Variable	Independent Variable	Unadjusted odds (95% CI)	Adjusted odds (95% CI)	p value
Nutritional Status 1 = Malnutrition 0 = Well nourished	Does not use soap after defecation	1.87 (1.19–2.93)	2.04 (1.27–3.29)	0.003
	Birthweight below < 2.5 kg	1.93 (1.11–3.34)	1.97 (1.11–3.47)	0.019
	Breastfeeding duration more than one hour	0.52 (0.33–0.81)	0.44 (0.27–0.70)	0.001
Adjusted for age of the child, gender of the child, water treatment before drinking, low monthly family income, schooling of mother, exclusive breastfeeding status of the child and food security status of the family				
Maternal education 1 = No education 0 = Educated	Low family income < USD 128	3.05 (1.81–5.16)	2.88 (1.70–4.89)	0.000
	Does not use soap for cleaning child after defecation	1.98 (1.13–3.27)	1.70 (0.99–2.93)	0.054
Adjusted for water treatment before drinking, use of soap for hand washing before food preparation, sharing of toilet with members of other households, nutritional status of the child and food security status of the family				

## Discussion

Our analysis of the household food access data showed that 83% of the selected households experienced food insecurity. In 2017, Global Food Security 13 reported similar results for Bangladesh. The National Micronutrient Status Survey of 2011–2012 14 also reported that 17% of the slum households in Bangladesh suffered from severe food insecurity. Household factors including food insecurity, inadequate or inappropriate complementary food, lack of breastfeeding practices and infectious illnesses were the leading factors associated with undernutrition in the children studied 15. The children in Slum S were at the bottom of the list in terms of nutritional status as well as household food security when the four slums were examined. Food insecurity is a critical variable in understanding the nutritional status of low‐income settlements.

Our study reported a higher rate of stunting (58%) than the national figure of 36% in Bangladesh Demographic and Health Survey 2014 4. However, researchers from the Malnutrition and Enteric Disease Study reported that 44% of the children aged under two years living in Slum B had impaired intestinal permeability characterised by abnormally high urinary lactulose, as indicated by the mannitol recovery ratios. These high recovery ratios (≥0.09) were considered to be an indication of environmental enteric dysfunction 16. As our study was conducted in four slums in Dhaka city, where hygiene practices were poor and there was a high level of contamination of complementary food, we can assume that environmental enteric dysfunction may have played an important role in relation to the high stunting rates among the slum dwelling children 17.

Mothers or caregivers in slums are less likely to have higher levels of education, and our finding was similar to an earlier observation 18. Key factors associated with the timely introduction of solid foods have been related to increasing maternal education and age 19. The present study demonstrated that a high proportion (59%) of mothers did not wash their hands before handling food and more than 80% prepared food at home. A survey conducted in Bangladesh observed that the educational status of the head of the household or respondent was positively associated with hand washing with soap 20. The poor personal hygiene practices of the mothers, especially hand washing practices before food preparation, may have been a contributing factor to the high contamination rate by pathogens. In this study, 99% of the mothers stored their children's food at room temperature, which may have led to rapid bacterial growth and proliferation in the food, particularly during the summer months. A study conducted in Tanzania suggested that a four‐hour time lapse between food preparation and consumption could result in a significant increase in total count of coliforms and *Enterobacteriaceae* 21. We found that nearly 45% of the food items served to the children were prepared eight hours before consumption (Data not presented).

*Bacillus cereus* was found in 48% of the food samples, and this level of contamination was much higher than a study conducted in urban Bangladesh where a rate of 33% was reported 5. *Bacillus cereus* causes food poisoning and diarrhoea by releasing enterotoxins and the bacterium grows well in food after cooking and cooling at less than 48°C 22. Around 30% of the food samples we collected were contaminated with *Escherichia coli* that are suggestive of direct faecal contamination 5, and this observation is not uncommon in developing countries 9. In relation to stunting, wasting or underweight, we did not find any specific association with microbial contamination, although our study was not powered to address this issue. The present study observed that all the malnourished children were equally affected by the food contaminated with yeast and mould and coliforms. However, this study did not find *Bacillus cereus* in the complementary food given to wasted children. Increased bacterial contamination of food could also be a sequel to the presence of a higher number of microorganisms in water, which is commonly used in preparing food for infants and young children. The family members in our study most commonly used tap water. According to the Bangladesh Urban Health Survey 2013, 65% of households used shared water and that finding corroborates our results. In that survey, the level of toilet sharing and inadequate sanitation practices was also very high (77%) 18. We found that 94% of the households shared toilets at the time of our study and only 24% families boiled their drinking water. According to the present study, water contamination with microorganisms was very common in urban slums. The 100% levels of contamination due to coliform, total aerobic bacterial count, *Staphylococcus*, yeast and moulds in this study may be indicative of problems with the water supply systems and microbial contamination at the point of use that exist at household levels in urban slums. These overcrowded slums have limited space between water sources and poorly maintained toilets, which allow microorganisms to drift from toilets to water sources and contaminate water sources by rapidly growing and multiplying enteric pathogens 9. Although Bangladesh has set a parameter that no *Escherichia coli* should be found in a 100‐mL sample of drinking water, this study found that 63% of the samples were contaminated with *Escherichia coli*. A study published in 2015 revealed that in urban areas of Bangladesh the most common cause of contamination in tap water was leaks from the water supply pipes 23, even though the piped water had an acceptable quality at the point of supply. Another study conducted in the Mirpur area of Dhaka city reported that 98% of the drinking water samples were contaminated with thermotolerant coliforms 24. During our microbiological analysis of the complementary foods, an increased number of food borne pathogens were detected at the point of sample collection. These counts were higher than the recommended bacterial count in international guidelines for the bacterial safety of complementary foods 21. Significant percentages of yeast and mould (86%) and coliforms (73%) were present in the complementary food samples we collected. Environmental yeasts are usually found in surface water and may be a problem of faulty water supply systems 25. As slums often suffer from a damp and unhealthy environment, food commonly contains yeast and mould contaminants.

We found that the children were offered more carbohydrate‐rich food, sacrificing protein heavy alternatives. Families may have also lacked the financial means to offer protein‐rich foods, contributing to the high rates of stunting among children living in slums. A study that focused on Peruvian and Kenyan children also found stunting among those who consumed little or no meat 26.

We could not record the birthweights in the present study, due to the high rates of home‐based births. Thus, only the mother's perception about her baby's birthweight served as a proxy indicator for assessing the low birthweight status of the newborn babies. According to the WHO 2011, birthweights of less than 2.5 kg are considered to be low birthweights 27. We found that 23% of the children were born with a perceived below average birthweight, which was much higher than that the data reported by Bangladesh Demographic Health Survey 2014 4 where 13% of children fell into that category. In this study, we found a high proportion of children, more than 60%, were malnourished in the four slums and a larger proportion of children were stunted (58%) compared to wasting (7%) and underweight (37%). These results suggest a high proportion of chronic undernutrition among the children living in slums. The results showed similarities with observations from 54 resource‐poor countries in Africa and Southeast Asia, where researchers reported a rapid drop in the height for age *Z* score during the first two years of life 28.

Information related to food preparation and hygiene practices was gathered through interviews and not by direct observations at household level. Microbial contamination of water was found to take place mostly at the storage or at the point of use, while at the collection point the water was often free from any contamination. Obtaining information on the microbial contamination of food and water from a higher number of households could have further strengthened our observations.

## Conclusion

Our study found that three main factors affected the nutrition and growth of children: poor environmental conditions; inadequate household hygiene practices and household food insecurity. All of these components indirectly affected the quality of the complementary food they received. As these factors are inter‐related, it would be wrong to focus on any individual factor or just some of them. Hygiene and sanitation interventions should be coupled with strengthening the knowledge and skills of mothers in food preparation and preservation techniques that could reduce the microbial contamination of complementary food and water.

In developing nations, access to safe water, environmental sanitation, proper management of human waste and hygiene facilities are achieved through a network of inputs from state agencies, nonprofit organisations, commercial providers and through the efforts of service consumers. We can therefore attribute the high levels of malnutrition observed among the slum children in our study to the inadequacy in the network of inputs from a diverse range of stakeholders who aim to assist Dhaka's slum dwellers. We suggest that integrated efforts are essential to create public awareness about hygiene and preparing clean food to ensure an overall improvement in the nutritional status of these children.

## Conflicts of interest

The authors have no conflicts of interest to declare.

## Funding

This study formed part of the EcoPoor, Institutions for Urban Poor's Access to Ecosystem Services: A Comparison of Green and Water Structures in Bangladesh and Tanzania research project. This work was supported by the Natural Environment Research Council (NERC) through its Ecosystem Services for Poverty Alleviation (ESPA) (http://www.espa.ac.uk) research programme (grant number NE‐L001616‐1). The International Centre for Diarrhoeal Disease Research, Bangladesh received core funding or unrestricted support for its studies from the Governments of Bangladesh, Canada, Sweden and the UK.

## Appendix A

### Case site selection

Slum B started about 25 years ago. By 1990, 2600 families had moved to this settlement where they were allocated a land area of 450 square feet, with a house of 96 square feet and promised a 99‐year land lease. It was divided into five blocks ‐ this study focused on one block (known as E block). The majority in this block had corrugated tin as roof or wall material but some were two‐storied concrete buildings or of mixed structures with more than 1000 households. The majority of residents there were tenants. Although the slum started as a government resettlement colony, afterwards it turned into a private slum and its management was under the local slum authority.

Slum K was a squatter settlement on government land. The majority of houses were tin‐shed, and the settlement was densely populated with over 800 households with an estimated population of 3500–4000. The settlement had a history of being recipient of developmental assistances on continued basis from nonprofit organisation.

Slum G was established by the Dhaka City Corporation for its low‐paid fourth‐class employees hence it was a government authorised slum. Approximately 3000–3500 people live in this area. Those employees were the support staff of Dhaka City Corporation like sweepers, security guards, ward boys, cleaners and gardeners and they were the residents, of Slum G, in approximately 750 families.

Finally, Slum S was located near the flood protection embankment (*Beri Badh*). Unlike the other settlements, Slum S consists of all privately owned rooms, some of which are constructed in two‐storied concrete buildings. Approximately 365 families, around 1460–1500 people were living in that slum. All living facilities were arranged and maintained by the house owners and the caretakers of the slum residents.

### Sampling size and sampling

For specimen collection, the study employed a simple, effective, easy‐to‐carry‐out, and inexpensive sampling strategy. In every slum, a central location such as a mosque, a low‐scale vegetable selling area, a school or a small makeshift bridge was selected as the starting point. Then, a direction was chosen, usually following a lane in the slum. The first household selected for the specimen collection was located in the closest, immediate proximity of the starting landmark. After that, listed households situated along the directional line from the starting point were visited, and specimens were collected only from first 18 households having specimen in adequate quantity for collection. As not all of the families visited necessarily had specimens for collection, more than the desired number of households was visited.

### Questionnaire and sample collection

The food security questionnaire consists of nine questions. The questions addressed the situation like anxiety and uncertainty; insufficient quality; and insufficient food intake and its physical consequences of all household members and did not distinguish adults from children and adolescents 29. The process of food collection, handling and feeding patterns was also recorded in data collection tools. A minimum of 100 g of food sample was collected directly from the feeding pot at household level, placed into a sterile plastic bag, and transported to the laboratory within two hours of collection maintaining a cold chain (+4 to +8°C).We have also collected 1 L of water in a sterile bottle from point of use from each enrolled family.

Slum = At present there is no agreed upon term to refer to where poor urban people usually live. The term slum is most commonly used but it usually has derogatory connotations and can suggest that a settlement needs replacement or can legitimise the eviction of its residents. However, it is a term which is difficult to avoid. In this study, we have used the term slum mainly for brevity and certainly not in any derogatory sense.

## Appendix B

### Microbial contamination of food and water

For enumeration of Coliforms and *Escherichia coli*, an aliquot of 25 g solid or 25 mL liquid food sample was mixed well with 225 mL of 0.1% peptone water. For enumeration of *Bacillus cereus*, coagulase‐positive *Staphylococcus* and yeasts and moulds, 100 μL aliquots of the same dilutions were inoculated onto selective plating media by spread plate technique and incubated at specified conditions. For isolation and identification of *Shigella* spp., *Salmonella* spp., *Vibrio* spp., and *Escherichia coli* the same amount of samples (25 g/25 mL) were taken for each of the organisms and mixed with selective enrichment media (225 mL). Only for *Salmonella*, pre‐enrichment of the samples was carried out at 37°C for 18–24 hour in buffered peptone water. The selective enrichment cultures were inoculated on to selective plating media. Typical colonies on culture plates were identified according to the standard procedures. For yeast and mould counting, culture plates were incubated in an upright position in undisturbed condition in the incubator until the plates were ready for counting. Identification of typical colonies on selective plates was carried out according to the standard procedures 5.

For qualitative analysis of *Vibrio cholera*, 50 mL of water sample was enriched in 25 mL triple strength alkaline peptone water and incubated for six hour at 37°C. Water sample of 50 mL was enriched in 25 mL triple strength selenite broth media and incubated for overnight at 37°C for examining *Shigella*,* Salmonella* and *Pseudomonas* spp. For total aerobic bacteria, 100 μL water samples were inoculated using the drop plate technique. For enumeration of faecal coliforms and *Escherichia coli*, 100 mL of water samples was filtered through 0.22 μm pore‐size membrane filter and the filters were placed on membrane faecal coliform and membrane Thermotolerant *Escherichia coli* agar plates. The selective enrichment cultures were inoculated on to selective plating media, and time and temperature for the organisms were maintained according to the standard laboratory procedure 30. For enumeration of yeasts and moulds, 100 mL of water sample was filtered through 0.22 μm pore‐size membrane filter and the filter was placed on Dichloran Rose Bengal Chlortetracycline agar plate 31. Typical colonies on culture plates were also identified according to the standard procedures 30.
